# Humoral and cellular immune response in patients of liver cirrhosis and immunocompetent recipient of ChAdOx1nCoV-19 Vaccine (Covishield)

**DOI:** 10.1007/s10238-023-01258-z

**Published:** 2024-01-27

**Authors:** Himanshu Dandu, Amit Goel, Manish Kumar, Hardeep Singh Malhotra, Harshita Katiyar, Monica Agarwal, Neeraj Kumar, Pragya Pandey, Shivani Rani, Geeta Yadav

**Affiliations:** 1 Department of Internal Medicine, King George’s Medical University, Lucknow, 226003 India; 2 Department of Hepatology, Sanjay Gandhi Post-Graduate Institute of Medical Sciences, Lucknow, 226014 India; 3 Department of Pathology, King George’s Medical University, Lucknow, 226003 India; 4 Department of Neurology, King George’s Medical University, Lucknow, 226003 India; 5 Department of Community Medicine, King George’s Medical University, Lucknow, 226003 India; 6 Department of Conservative Dentistry and Endodontics, King George’s Medical University, Lucknow, 226003 India

**Keywords:** COVID-19, Vaccine, T cell, Memory cell, Flow cytometry, Cirrhosis

## Abstract

Despite the effectiveness of COVID-19 vaccination in reducing the severity of the disease, the demand for booster is increasing in vulnerable populations like elderly and immunocompromised individuals especially with each new wave of COVID-19 in different countries. There is limited data on the sustained immunity against COVID-19 in patients with liver cirrhosis. The study was aimed to compare the T cell and humoral immune response after 1 year of ChAdOx1nCoV-19 Vaccine in patients with liver cirrhosis and healthy health care workers (HCW). This was a prospective observational study including 36 HCW, 19 liver cirrhosis patients and 10 unvaccinated individuals. Anti-SARS-CoV-2S antibody, neutralizing antibody and memory T cell subsets were evaluated by ELISA and flow cytometry, respectively, in all three groups after 1 year of initial vaccination. Compared to HCW and unvaccinated individuals, liver cirrhosis patients had significantly depleted T cells, although CD4:CD8 + T cell ratio was normal. Both cirrhotic patients and HCW developed memory T cell subset [effector memory RA (*P* = 0.141, *P* < 0.001), effector memory (*P* < 0.001, *P* < 0.001), central memory (*P* < 0.001, *P* < 0.01), stem cell memory (*P* = 0.009, *P* = 0.08) and naïve (*P* < 0.001, *P* = 0.02)] compared to unvaccinated unexposed individuals of CD4 + T and CD8 + T, respectively. However, among HCW and cirrhotic group no difference was noted on central memory and stem cell memory cells on T cells. Patients with liver cirrhosis developed comparable memory T cells after vaccination which can evoke sustainable immune response on reinfection. Therefore, additional vaccine doses may not be necessary for cirrhosis patients.

## Introduction

The COVID-19 pandemic has continued for more than 2 years infecting half of the world population either by natural infection or by vaccination. More than 772 million people have been infected and about 6.98 million deaths have occurred owing to SARS-CoV-2 infection as of December 2023. A total of 13.5 billion vaccine doses have been administered till November 25th, 2023 [1]. The available vaccines in India at the time of study were the Astra Zeneca (ChAdOx1 nCoV-19) vaccine (Covishield, Oxford/Astra Zeneca COVID-19 AZD1222, Serum Institute of India), a non-replicating viral vector vaccine (NRVV) and Bharat biotech BBV152 (Covaxin [BBV152]) containing inactivated whole virus; both of which required two doses for effective action. Covishield vaccine uses modified viral vector platform of chimpanzee adenovirus (ChAdOx1) that permits it to transfer spike protein of COVID-19 virus into human cells [2]. Study based on the in vitro live-virus neutralization and T cell immune responses to the spike protein showed that the efficacy of two doses of Covishield vaccine against moderate-to-severe COVID-19 was ~ 81.5% [3]. Multiple studies have quoted that after natural SARS-CoV-2 infection or vaccination, vigorous T cells response is responsible for production of anti-spike Neutralizing antibodies against multiple viral epitopes like spike Anti-receptor binding domain (RBD) & N-Terminal domain (NTD) [4–6]. The mRNA and adjuvant vaccines promote intracellular production of spike protein which is presented by antigen presenting cells to naïve T cells (both CD8 + and CD4 + T cells) causing activation and differentiation of T cells into effector cells and different subsets of memory T cells [7]. Vaccines function by triggering an immune response, leading to the development of immunological memory, which subsequently provides protection against infection or disease. These memory cells which are once invoked either by natural infection or by vaccination can persists even after decades. The interplay of this cellular and humoral response is essential for effective immunity [8, 9].

Vaccination against COVID-19 has reduced the severity of the disease, yet we are still facing waves of COVID-19 infection like the recent surge in China and India [10, 11]. Demand for booster dose is brought up with each wave of COVID-19, especially in vulnerable population like elderly, cancer patients and immunocompromised population [12]. Studies have monitored the titer of neutralizing antibody at different time interval to check for adequate humoral immune response in healthy individuals [13–15]. Few studies have mentioned waning effect of humoral response at 6 months of vaccination by measuring IgG neutralizing antibodies (NAb) levels and proposed that reactivity and durability of cellular response (CD8 + cells) can prevent severe disease against viral variant even when they escape from Nab. Equally, T cell response at different time period has also been studied in healthy individuals [4, 16, 17]. But there is paucity of data in immunocompromised individuals for sustained immune response especially with regards to T cell-based immunity. Cirrhosis is associated with impairment of innate and adaptive immune system leading to acquired immunodeficiency. Both B and T cell mediated immunity is hampered in form of B and T cell depletion and dysfunction because of multifactorial causes like impaired production, proliferation and increased apoptosis [18, 19]. Hence, we selected patients of cirrhosis as immunocompromised population in our study. We studied different subset of T cell memory cells along with antibody titer at baseline and at 1 year duration in vaccinated healthy individuals and patients with liver cirrhosis at 1 year to observe any difference in immune response.

## Material and methods

### Study design and population

This was a prospective longitudinal cohort study done between May 2021 and June 2022 involving 36 health care workers (HCW) and 19 cirrhosis patients at King George’s Medical University and Sanjay Gandhi Postgraduate Institute of Medical Sciences, Lucknow. Blood samples of 10 healthy unvaccinated (and unexposed as per history) individuals were obtained from rural area and they were referred for vaccination at primary health care center. The normal healthy HCW were defined as without any co-morbidity or medication. Patients were selected for the cirrhotic group based on clinical, biochemical and radiological finding along as well as evidence of portal hypertension and AST–Platelet ratio index. Hepatic decompensation was determined by the presence of ascites or hepatic encephalopathy in cases of hepatitis B-related liver cirrhosis, or by a markedly raised serum bilirubin level along with prolonged prothrombin time or international normalized ratio > 1.5 [20]. Venous blood was collected from all participants before receiving the first dose (day 0) and before booster dose (day 270 ± 14 after second dose or ≅ 1 year after first dose). The time interval between first and second dose was 3 months as per government protocol at the time of vaccination. Samples were collected in plain and EDTA vial for anti-SARS-CoV-2 antibody and flow cytometry assay, respectively. All participant signed written informed consent form and study was approved by institutional ethical committee.

### Serological assay

Anti-receptor binding domain (RBD) or anti-Spike antibody titer and neutralizing antibody (NAb) were measured in stored serum sample by double-antigen sandwich enzyme-linked immunoassay (ELISA) through Elecsys® Anti-SARS-CoV-2 S (Roche Diagnostics GmbH, Germany) and SARS-CoV-2 Neutralizing Antibody competitive ELISA Kit (Invitrogen, Thermo-Fischer), respectively. For anti-RBD assay, sample was incubated with biotinylated and ruthenylated RBD antigens, forming double antigen sandwich immune complexes (DAGS) followed by addition of streptavidin-coated microparticles. After transfer to the measuring cell, microparticles were magnetically captured and detected by electro-chemiluminescent signal proportional to the antibody titer. The Elecsys® Anti-SARS-CoV-2 S antibody titer was expressed as U/mL and value > 0.8 U/mL was considered positive (quantitation limit of 0.40–250 U/mL). For SARS-CoV-2 Neutralizing Ab assay, samples were applied to wells coated with RBD protein, incubated for 30 min. Neutralizing antibodies in the sample, binding specifically to RBD, obstructed its interaction with biotinylated ACE2 added for 30 min post-wash. Streptavidin conjugated horseradish peroxidase (HRP) signal inversely correlated with the specific neutralizing antibody quantity. For SARS-CoV-2 Neutralizing Antibody, samples with more than 20% calculated neutralization were considered positive.

### Flow cytometry assay

**Fig. 1:**
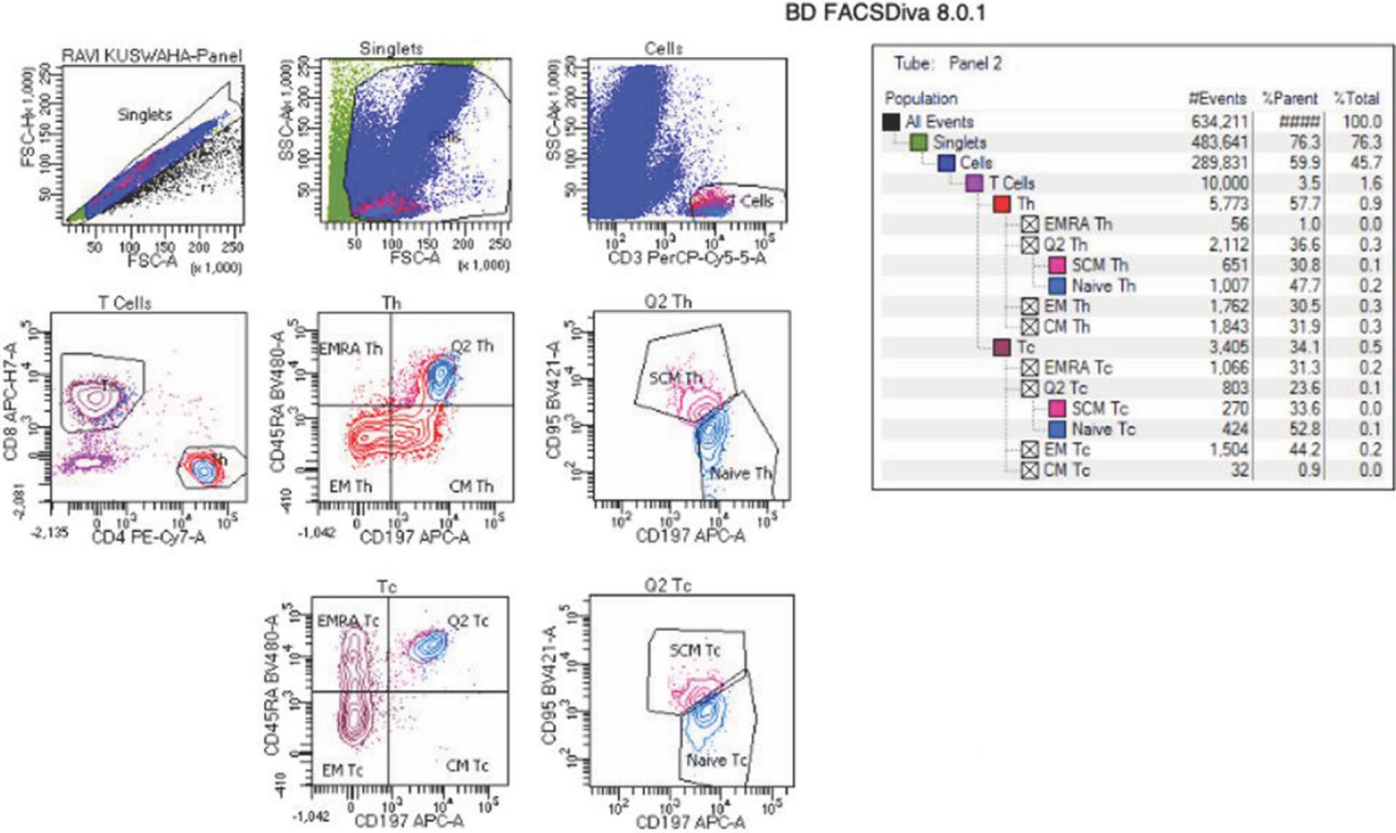
Flow plot showing sequential gating strategy; singlets followed by viable cells followed by T cells (SSC vs CD3), followed by CD4 + T and CD8 + T cells (CD4 + vs CD8 +). From either CD4 + or CD8 + T cell gate, central memory (CM), effector memory (EM) and effector memory RA (EMRA) subsets were identified based on differential expression of CD45RA and CD197 (CCR7). From the CD45RA + CD197 + gate, CD95 + stem cell memory and CD95 − naïve T cells were further identified

The sample preparation for flow cytometric analysis was done by stain-lyse-wash protocol. The antibodies utilized in the assay were procured from Becton Dickinson (BD) Biosciences, San Jose, California, USA. The fluorochrome and clones of antibody used were CD3 PerCP-Cy5.5 (SP34-2)/CD4 PE-Cy7 (SK3)/CD8 APC-H7 (SK1)/CD197 APC (2-L1-A)/CD95 BV421 (DX2)/CD45RA BV480 (H1100)/CD27 PE (M-T271) in the memory cell tube. A blank tube without antibody was also run. While FACS Canto II flow cytometer, BD Biosciences was used for sample acquisition, data analysis was performed using FACS Diva version 8 software. At least 10,000 CD3 + T cells were acquired in each case to ensure adequate number of subset population to be studied. Based on light scatter properties, lymphocytes were identified. From this population, CD3 + total T cells as well as CD4 + and CD8 + subsets were subsequently defined. On the basis of differential expression of CD45RA and CD197 (CCR7), central memory (CM), effector memory (EM) and effector memory RA (EMRA) subsets were identified on both helper and cytotoxic cell. From the CD45RA + CD197 + gate, CD95 + stem cell memory and CD95- naïve T cells were further identified. The gating strategy has been depicted in Fig. 1.

### Statistical analysis

Normality assessment of data was done with Shapiro Wilk test. Continuous data was expressed as mean (with standard deviation) or median (with range) with respect to its distribution. To compare the expression of antibodies, Mean fluorescence intensity (MFI) was used. Nonparametric assessments were used to compare the different groups; 2-independent sample analysis was done using Kolmogorov–Smirnov *Z* test while > 2-independent sample analysis was done by Kruskal–Wallis ANOVA with post-hoc pairwise comparisons. All assessments were 2-tailed and a *P* value of < 0.05 was taken as significant; all values < 0.001 were expressed as < 0.001. Data were analyzed with IBM SPSS package for Windows, Version 24.0, Armonk, NY.

## Results

### Demography and hematological parameters

We evaluated 19 patients of cirrhotic liver disease, 36 normal health care workers and 10 unvaccinated healthy individuals at 1 year duration after initial dose of vaccination. All individuals received two doses of ChAdOx1nCoV-19 vaccine. The demographic and laboratory findings of study population are described in Table 1.

**Table 1:** Demographic and laboratory parameters of study population

Variables (Median range)	Cirrhotic patients (*n* = 19)	Health care workers (*n* = 36)	Unvaccinated (*n* = 10)	*P* value
Age (years)	52 (44–67)	49 (41–56)	48 (39–55)	0.08
Male:Female ratio	14:5	22:14	1:9	0.003
*Co-morbidity*				
Diabetes mellitus	15.79% (3/19)	0	0	
Hypertension	84.21% (16/19)	0	0	
Both	26.31% (5/19)	0	0	
*Underlying etiology*				
Alcohol	15.79% (3/19)			
Hepatitis B	10.52% (2/19)			
Hepatitis C	52.63% (10/19)			
Cryptogenic/NASH	10.52% (2/19)			
Others	15.79% (3/19)			
*Cirrhosis*				
Compensated	84.21% (16/19)			
Decompensated	15.79% (3/19)			
*Child–Turcotte–pugh (CTP)*				
CTP score	8 (5–8)			
CTP Class A	15/19			
CTP Class B	4/19			
CTP Class C	0/19			
*Laboratory parameters*				
Hemoglobin (g/dL	13.2 (8.4–14.3)	13.5 (11.0–15.6)	13.0 (9.4–14.8)	0.408
Total leukocyte count (× 10^9^/L)	6.4 (4.1–8.7)	7.05 (4.2–12.4)	5.36 (5.8–9.79)	0.001
Platelet count (× 10^9^/L)	70 (100–220)	189 (70–279)	99.5 (74–890)	0.855
Absolute neutrophil count (× 10^9^/L)	5.9 (3.5–7.1)	5.5 (3.2–8.1)	4.6 (3.5–7.70)	0.000
Absolute lymphocyte count (× 10^9^/L)	2.5 (1.7–5.1)	3.4 (1.7–3.7)	4.3 (2.8–6.5)	0.004
International normalization ratio (INR)	1.3 (1.08–1.90)			
Serum creatinine (mg/dL	0.9 (0.7–1.3)			
Serum bilirubin total (mg/dL	1.4 (0.2–4.2)			
Serum protein (gm/dL)	7.6 (6.1–8.9)			
Serum albumin (gm/dL)	3.8 (3.4–5.1)			
Serum alanine transaminase (IU/L)	37 (25–173)			
Serum aspartate aminotransferase (IU/L)	49 (21–280)			
Serum alkaline phosphatase (IU/L)	112 (60–211)			

Data are presented as the median (range), or n (%). NASH (non-alcoholic steatohepatitis), CTP (Child–Turcotte–pugh)

### Anti-RBD antibody and neutralizing antibody at baseline and at 1 year

**Fig. 2:**
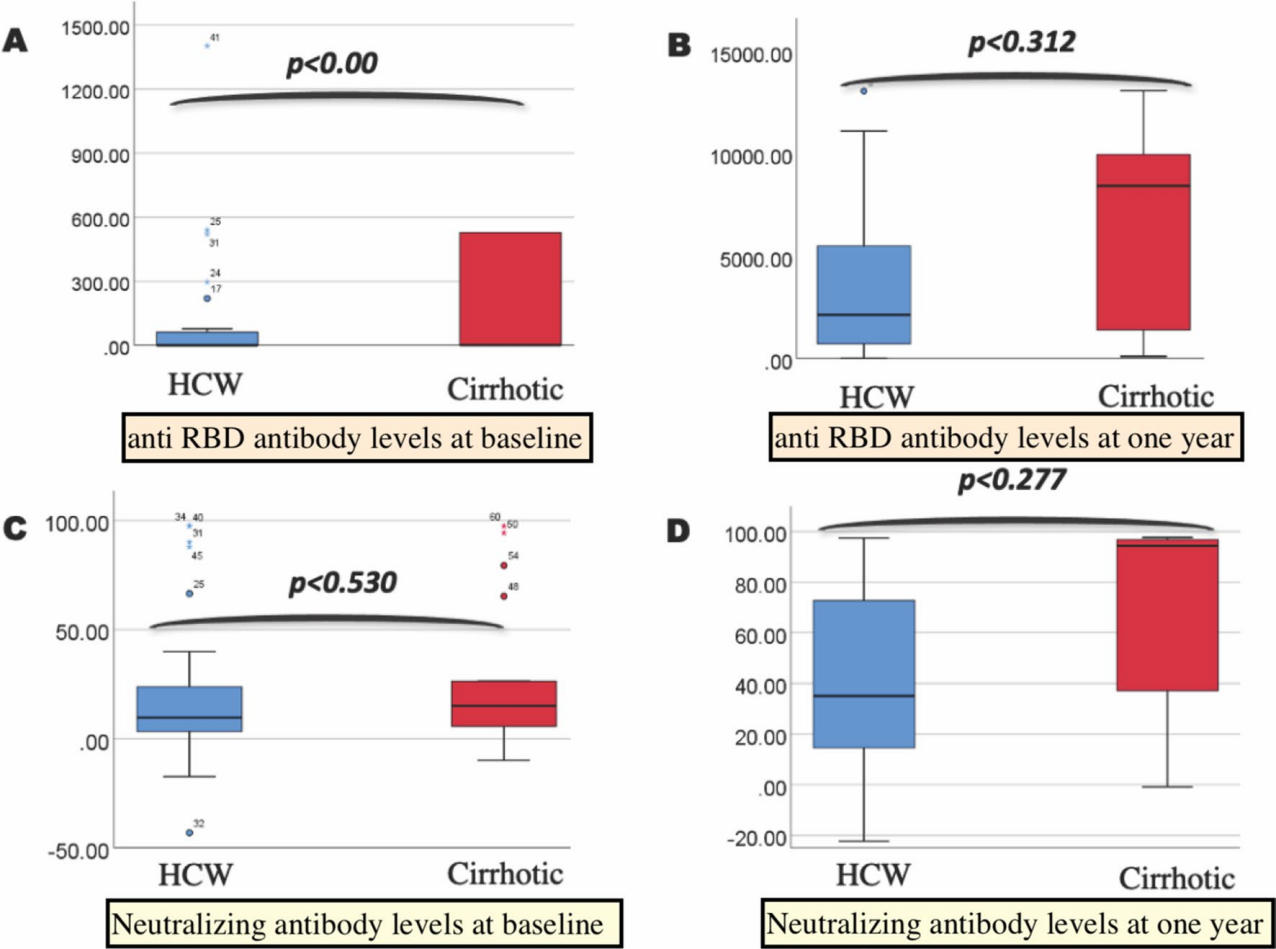
**a** Box plot comparing the anti-RBD antibodies levels at baseline, **b** Box plot comparing the anti-RBD antibodies levels after 1 year of vaccination, **c** Box plot comparing the neutralizing antibodies levels at baseline, **d** Box plot comparing the neutralizing antibodies levels after 1 year of vaccination among health care workers and cirrhotic patients

The baseline anti-RBD antibody (U/mL) and neutralizing antibody (%) titer in cirrhotic and HCW was 0.675 (0.4–10,478) and 54.7 (0.04–1403.0) and 11.2 (− 17.39 to 97.35) and 15.02 (− 10.04 to 97.3), respectively. Three patients in cirrhotic group had higher levels of anti-RBD antibody and NAbs levels even before vaccination which could be due to asymptomatic natural SARS-CoV-2 infection. All participants in cirrhotic and HCW group showed development of antibodies and the median titers after 1 year were 2132 (11.09–42594) and 8444 (101.6–23,044) and 35.57 (− 22.29 to 97.4) and 94.3 (− 0.8 to 97.6), respectively. There was no difference between anti-RBD and neutralizing antibody titer at 1 year between cirrhotic and HCW group (*P* = 0.312, *P* = 0.277). The unvaccinated group had < 0.4U/mL and < 20% anti-RBD antibody (U/mL) and neutralizing antibody levels (Fig. 2).

### Memory T cell response at 1 year

Significant depletion of T cells was noted in cirrhotic patients (5.8 [0.5–20.1]) as compared to HCW (13.1 [1.6–31.3]) and unvaccinated individuals (12.3 [7.8–19.6]; (*P* = 0.001 &nd *P* < 0.001), respectively. However, the ratio of CD4 + T to CD8 + T cells was not altered in any group. By analyzing expression of CD45RA and CD197 (CCR7), subsets of central memory (CM), effector memory (EM) and effector memory RA (EMRA) were identified in both CD4 + or CD8 + T cell. From the CD45RA + CD197 + gate, CD95 + stem cell memory and CD95- naïve T cells were further identified. Significant difference was noted in various memory subsets [EMRA (*P* = 0.141, *P* = 0.000), EM (*P* < 0.001, *P* < 0.01), CM (*P* < 0.001, *P* < 0.001), SCM (*P* = 0.009, *P* = 0.08) and naïve (*P* = 0.000, *P* = 0.02)] of CD4 + T and CD8 + T in all three group, respectively (Table 2).

**Table 2:** Median of mean florescence intensity (MFI) of different memory CD4^+^ T and CD8^+^ T cells

Variable median (range)	Cirrhotic (*n* = 19)	Health care workers (*n* = 36)	Unvaccinated (*n* = 10)	*P* value
Total events (million)	4.07 (0.49–10.60)	3.86 (0.27–12.54)	4.54 (3.63–6.98)	0.300
T cells (% of total viable cells)	5.8 (0.5–20.1)	13.1 (1.6–31.3)	12.35 (7.8–19.6)	0.001
CD4 + (%T cells)	57.5 (17–62.1)	48.7 (38.1–68.3)	44.4 (34.3–61)	0.132
CD8 + (%T cells)	33.1 (19.3–54.5)	38.3 (32–67.4)	35.45 (4–54.9)	0.074
CD4 + EMRA (% of CD4 +)	1.8 (0.4–40.2)	3.7 (0.5–69.7)	5.1 (2.8 -91.7)	0.107
CD4 + EM (% of CD4 +)	35.4 (1.0–48.5)	30.6 (11.5–60.2)	94.3 (4.5–96.9)	0.001
CD4 + CM (% of CD4 +)	34.6 (0.0–63.6)	32.2 (14.2–58.9)	0.3 (0.1–0.2)	0.001
CD4 + SCM (% of CD4 +)	4.86 (1.35–29.32)	4.32 (1.5–38.9)	0.215 (0–1.85)	0.001
CD4 + Naive (% of CD4 +)	14.98 (4.1–26.8)	14.46 (6.5–37.83)	0 (0–0.5)	0.001
CD8 + EMRA (% of CD8 +)	51.3 (14.9–80.9)	37 (7.3–68.7)	6.3 (2.7–25.5)	0.001
CD8 + EM (% of CD8 +)	18.8 (2.7–44.2)	28.9 2 (7.3–45.7)	93 (73.4–97)	0.001
CD8 + CM (% of CD8 +)	3.9 (0.4–10.2)	3.4 (1.3–12.6)	0.2 (0.1–0.4)	0.001
CD8 + SCM (% of CD8 +)	4.5 (4.0–60.6)	3.1 (1.8–47.04)	0.185 (0.3–1.16)	0.001
CD8 + Naive (% of CD8 +)	7.79 (0.9–83.4)	6.71 (2.59–32.04)	0.035 (0–0.5)	0.001

Mean florescence intensity is shown in median (range), EMRA (effector memory RA), EM (effector memory), CM (central memory), SCM (stem cell memory)

**Fig. 3:**
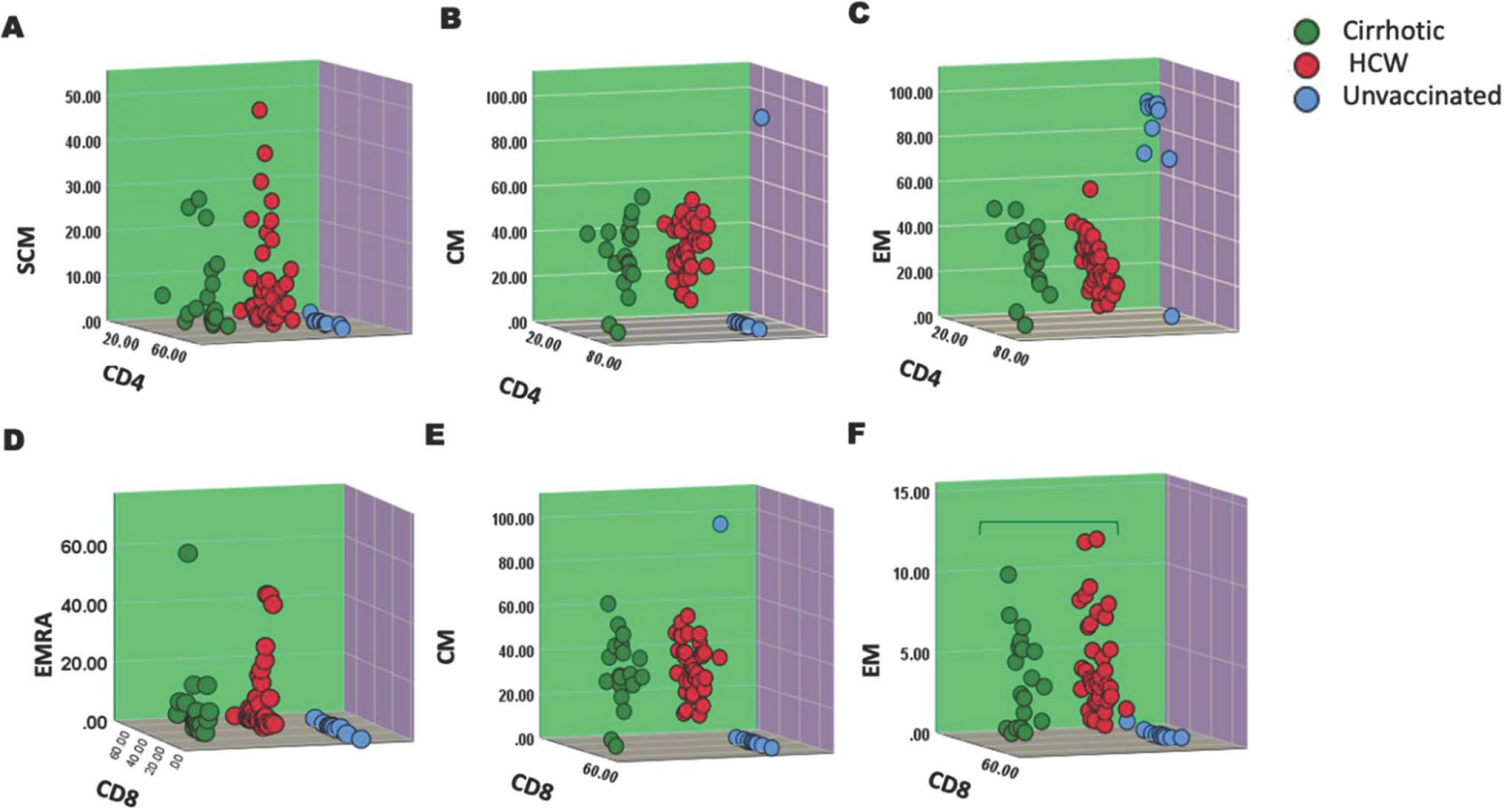
Three-dimensional scatter plot of mean frequency of distribution of memory T cells on CD4 + and CD8 + T cells among cirrhotic, health care workers and unvaccinated individuals

On post-hoc analysis, directed at cirrhotic versus HCW, only T cell distributions were found to be statistically different in the two groups (*P* < 0.001; Kolmogorov–Smirnov *Z* = 2.119). The various memory cells subsets in cirrhotic and HCW group did not show any difference, though there was significantly raised in comparison to unvaccinated individual (Fig. 3).

**Fig. 4:**
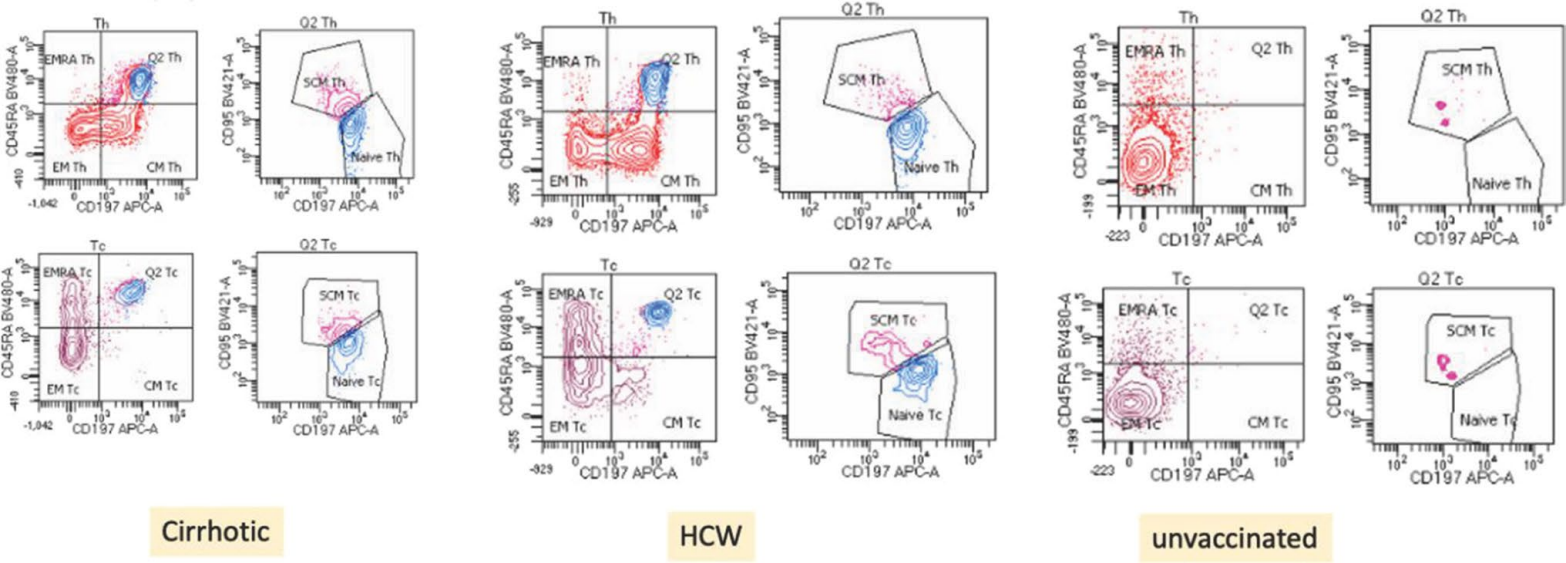
Flow plot showing frequency of expression of different subset of memory cells (both CD4 + and CD8 + T cells) in cirrhotic, health care workers and unvaccinated individuals

CM and SCM are responsible for long-term maintenance of memory cells which were absent in unvaccinated individuals as they were not vaccinated. All HCW and 89.47% cirrhotic patients developed CD4 + T_CM_. 63.88% HCW and 47.36% cirrhotic patients developed CD4 + T_SCM_. 36.11% HCW and 36.84% cirrhotic patients developed CD8 + T_CM_. 41.66% HCW and 42.10% cirrhotic patients developed CD8 + T_SCM._ There was no difference between memory cells on T cells between vaccinated cirrhotic patients and vaccinated HCW (Fig. 4).

## Discussion

This study compares the cellular and humoral response in cirrhotic and immunocompetent HCW after 1 year of receiving ChAdOx1nCoV-19 Vaccine (Covishield). All individuals received two doses of vaccine. The study found that both groups showed had similar seroconversion as denoted by titers of anti-RBD and neutralizing antibodies (*P* = 0.312, *P* = 0.277). Though the T cells were significantly reduced in cirrhotic patients, CD4:CD8 T cell ratio was normal in cirrhotic patients. Both CD4 + and CD8 + T cell subsets developed comparable CM and SCM in cirrhotic and HCW, which is suggestive of similar cellular immune response.

Due to the ongoing fear of developing fatal complication of SARS-CoV-2 infection, immunocompromised patients, like HIV, cancer, and patients with solid organ transplant, need special care and precaution [21]. Lee et al. in a meta-analysis of 83 studies using mRNA, VVV and inactivated whole virus vaccine in immunocompromised patients (solid cancer, inflammatory diseases, HIV & transplant recipients) found that after first dose of vaccination seroconversion rate was half in immunocompromised patients compared to the immunocompetent one. Second dose of vaccine was associated with consistently improved seroconversion in all groups with lesser magnitude in transplant individuals. Based on the above data authors advocated for the targeted intervention (third or booster dose) in immunocompromised patients [22]. Costiniuk et al., in their study on HIV patients, found that anti-RBD and anti-S antibody levels were maintained in 92% patients after 6 months and in 100% patients after 1 month of third dose and suggested timely booster administration in HIV patients for improved immune response [23]. Studies have shown reduced seroconversion in immunocompromised patients [24]. Therefore, many European countries recommend booster (third) dose for immunocompromised individuals; even some countries advocate extended (forth dose) or yearly dose in susceptible and vulnerable population with different cut-off for age, citing the waning effect of vaccination over time [25].

Effective vaccination develops both humoral and cellular response that can elicit adequate immune response on reinfection. Effector memory (EM) T cells serves as immediate effector function at frontline barriers and recall responses are mediated by CM and SCM even in the absence of neutralizing antibodies [26]. Crucial role of T cell immune response in SARS-CoV-1 infection has been clearly studied in animal models. On long-term follow-up, only 8.69% (2/23) patients of SARS-CoV-1 patients have shown detectable levels of IgG at 6 years indicating reduce humoral immunity over time whereas durable memory T cells were detected against SARS-CoV-1 at even > 10 years after infection. These findings may help us to understand the potential cross-reactivity with existing SARS-CoV-2 and effectiveness of T cell memory cells [7].

Cirrhosis is associated with dysfunction of both innate and adaptive immune system and is a state of acquired immunodeficiency [18]. It is characterized by expression of co-stimulatory and inhibitory immune checkpoints on T cells thereby casing prolonged exhaustion of adaptive immune response [27]. Cirrhotic patients developing COVID-19 infection seem to have a worse outcome than those otherwise [28]. Data on the duration of vaccine efficacy toward COVID-19 in patients with cirrhosis are sparse. The literature suggests that the underlying immune dysfunction in cirrhosis may lead to suboptimal response to vaccination as seen with hepatitis B and pneumococcal vaccines [29, 30]. However, recent studies have shown an improved outcome, reduced hospital stay and mortality in patients with cirrhosis undergoing COVID-19 vaccination.

Our study shows concordance in equal seroconversion in cirrhotic and immunocompetent HCW in developing comparable titers of anti-RBD and neutralizing antibodies after 1 year of initial dose of vaccination, however, differ in concept of booster dose requirement as persistent cellular immune response in form of induced stem cell memory and central memory were intact and similar in both cirrhotic and HCW which upon antigenic exposure have ability to mediate adequate immune response.

Several studies have revealed the disparity between B cell and T cell mediated immune response in recipients of kidney transplant. It has been observed that prevalence of S-protein specific CD4 + T in transplant recipient is comparable to healthy control despite a lower humoral response after two dose of vaccination [31]. Additionally, research has demonstrated that cytokines are produced by S-protein-reactive T cells on ex vivo antigenic stimulation indicating that they can mediate antiviral response activity and safeguard patients against severe COVID-19, despite the lack of antibodies. Consequently, relying solely on antibody detection to assess vaccination response may underestimate existing antiviral protection [32].

Feng et al. in their randomized control trial of vaccine efficacy study determined a binding antibody unit of 264 or a pseudo-virus neutralization assay titer of 26 IU/ml corresponded to 80% efficacy against symptomatic COVID-19 though they could not determine threshold for asymptomatic infections in their study [32]. Therefore, more studies with a long-term follow-up on larger population (including vulnerable and different spectrum of immunocompromised individuals) are required to define the reliable vaccine specific correlates of protection as in Hepatitis B.

To summarize, while studies in immunocompromised groups, including solid organ transplant recipients, HIV patients, and those with solid cancer, suggest the need for extra vaccine doses, our study indicates that both healthy individuals and those with cirrhosis generate comparable levels of memory T cells post-vaccination. This suggests robust and lasting immunity, possibly negating the necessity for additional vaccine doses in cirrhosis patients, contrary to recommendations for other immunocompromised groups.

One of the strengths of the study is that it examined the detailed cellular and humoral immune responses of cirrhotic patients with varying degrees of liver disease severity and causes, over a 1-year period after receiving the ChAdOx1nCoV-19 vaccine. However, a limitation of the study is the small sample size, with fewer patients in the CTP class C category.

## Data Availability

The datasets used and/or analyzed in the current study are available from the corresponding author on reasonable request.
